# Supplementary material for: Microbiome research in general and business newspapers: How many microbiome articles are published and which study designs make the news the most?
Source: PLoS One. 2021 Apr 9;16(4):e0249835. doi: 10.1371/journal.pone.0249835 (PMC8034714; doi:10.1371/journal.pone.0249835)
Supplement: S1 File — (DOC) [file pone.0249835.s001.doc]

**S1 File. Factiva search filters and phrases.**

Search filters:

- Default exclusion filters activated in the Factiva configuration.
- Filter for the location and elimination of duplicates: “identical”.
- Filter for sorting results: “oldest first”.

Complete phrase used in Spanish:

(microbioma* or metagenoma* or microbiota* or microflora or “microbios que cuidan nuestra salud” or “ecosistema interior” or “microbios salvadores” or “bacterias en el cuerpo” or “bacterias comensales” or “microbios buenos” or “bacterias saludables” or “bacterias buenas” or “bacterias beneficiosas” or “bacterias del cuerpo” or “bacterias que llevamos dentro” or “flora microbiana” or “flora bacteriana” or “flora microbial” or “bacterias amigas” or “bacterias de nuestro organismo” or “bichitos de nuestro organismo” or “microorganismos buenos” or “bacterias de la flora” or “hongo amigo” or “virosfera” or “viroma” or bacteria* gastrointestinal* or “vecinos de tu intestino” or “bacterias del sistema digestivo” or “especies bacterianas intestinales” or “bacterias de las tripas” or “inquilinos de tu intestino” or “flora del intestino” or “flora intestinal” or “flora colónica” or “bacterias intestinales” or “bacterias del intestino” or “bacterias en el intestino” or “bacterias de tu intestino” or “microorganismos intestinales” or “microbios intestinales” or “microbios del intestino” or “micobiota intestinal” or “comunidad viral del intestino” or “bacterias bucales” or “bacterias orales” or “flora oral” or “microbios de tu boca” or “microbios de tu estomago” or “flora de los pliegues” or “flora cutánea” or “microbios de la piel” or “bacteria de la piel” or “bacterias de nuestra piel” or “flora vaginal” or “microflora vaginal” or “microbios de la vagina” or “bacterias vaginales” or “bacterias en la vagina” or “universo microbiano en cada vagina” or “flora mamaria” or “flora de la madre” or “flora materna” or “bacterias de la leche materna” or “bacterias maternas” or “adn bacteriano de la madre” or “bacterias del pene” or ”bacterias de los ojos” or “bacteria que habita dentro de la nariz”) and la=es and sc=**

** **publication code**:

- *El País*: **paisn** (print edition) and **paisco** (digital edition).
- *Expansión*: **expnsi** (print edition) and **excom** (digital edition).

Complete phrase used in English:

(microbial ecosystem or microbial communities or body’s microbial garden or universe of us or microbiome* or metagenome* or mycobiome* or virome* or “viral flora” or microbiota* or microflora* or “bacterial communities we live with” or commensal bacteria* or native microbe* or commensal microbe* or “body’s microbial community” or friendly germ or beneficial bug* or “healthful bacteria” or “healthy bacteria” or “microbial inhabitants” or resident microbe* or “microbes inside us” or “good bacteria” or “beneficial bacteria” or “good microbes” or “good bugs” or “healthy flora” or “microbial flora” or “bacterial flora” or “microbial inhabitants” or “beneficial gut microbes” or “germs in your gut” or “good germs that live in your gut” or “beneficial gut germs” or “gut communities” or “gut bugs” or “microorganisms living in a person’s gut” or “microbes found in human intestines” or “bacteria that dwells in our intestinal tract” or “gut friendly bacteria” or “gut flora” or “intestinal flora” or “intestinal bacteria” or gut bacteri* or “gut-dwelling bacteria” or gut microb* or “bacteria in the gut” or intestinal microbe* or “intestinal colonies” or “bacteria colonizing our gut” or “microorganisms that live in the gut” or bacteria w/2 human gut or bacteria w/6 human gut or bacteria w/3 intestine* or “species in the gut” or “microbes in the gut” or “faecal flora” or “fecal flora” or “stool bacteria” or “skin bacteria” or “vaginal bacteria” or “vaginal flora” or “maternal flora” or “bacteria from the mother” or “maternal microbes”) and la=en and sc=**

** **publication code**:

- *The New York Times*: **nytf** (print edition) and **(nytb or nytfeed)** (digital edition).
- *The Times*: **t** (print edition) and **timeuk** (digital edition).
- *The Wall Street Journal*: **j** (print edition) and **wsjo** (digital edition).
- *Financial Times*: **ftft** (print edition) and **ftcom** (digital edition).
