# Supplementary material for: Microbiome research in general and business newspapers: How many microbiome articles are published and which study designs make the news the most?
Source: PLoS One. 2021 Apr 9;16(4):e0249835. doi: 10.1371/journal.pone.0249835 (PMC8034714; doi:10.1371/journal.pone.0249835)
Supplement: S2 File — (DOC) [file pone.0249835.s002.doc]

**S2 File. PubMed search filters and phrases.**

The general search equation for retrieving microbiome papers in PubMed was as follows:

("microbiota"[TW] OR "microbiotas"[tw] OR "microbiotae"[tw] OR “microbiota”[Mesh] OR "metagenome"[TW] OR "metagenomics"[TW] OR "metagenomic"[tw] OR "metagenomes"[tw] OR "microbial community"[tw] OR "microbiome"[tw] OR "gut microflora"[tw] OR "gastrointestinal flora"[tw] OR "gut flora"[tw] OR microflora[tw] OR microflorae[tw] OR microfloras[tw] OR “intestinal microflora”[tw] OR "intestinal flora"[tw] OR "intestinal floras"[tw] OR “enteric bacteria”[tw] OR “enteric bacterium”[tw] OR "intestinal microbe"[tw] OR "intestinal microbes"[tw] OR "gut microbe"[tw] OR "gut microbes"[tw] OR “beneficial microbe”[tw] OR “beneficial microbes”[tw] OR “vaginal flora”[tw] OR “vaginal floras”[tw] OR mycobiome[TW] OR mycobiota[tw] OR mycobiotas[tw] OR “gut virome”[tw] OR “commensal bacteria”[tw] OR “commensal bacterium”[tw] OR "commensal microbe"[tw] OR "commensal microbes"[tw] OR "gut commensal"[tw] OR “gut commensals”[tw] OR “commensal microorganism”[tw] OR “commensal microorganisms”[tw] OR “gut microbial”[tw] OR “gut bacterial”[tw] OR “intestinal microbial”[tw] OR “host microbial interactions”[tw] OR “host microbial interaction”[tw] OR “human microbial”[tw] OR “human microbes”[tw] OR “human microbe”[tw] OR “gut ecology”[tw] OR “bacterial flora”[tw] OR “bacterial floras”[tw] OR “bacterial florae”[tw] OR “fecal transplantation”[tw] OR “faecal transplantation”[tw] OR “fecal transplantations”[tw] OR “fecal transfer”[tw] OR "prebiotics"[MeSH Terms] OR "prebiotics"[TW] OR "prebiotic"[tw] OR "synbiotics"[TW] OR "synbiotic"[tw] OR "probiotical"[tw] OR "probiotics"[TW] OR "probiotic"[tw] OR "postbiotic"[tw] OR "postbiotics"[tw] OR “microbiota-gut-brain axis”[tw] OR “dysbiosis”[tw] OR “dysbacteriosis”[tw] AND (english[la] OR spanish[la]) AND (2007:2019[pdat]))

For retrieving microbiome papers segmented by study design we searched PubMed for the above search equation connected by the following filters through ‘AND’ operator in search engine.

| Systematic reviews of randomized controlled trials in humans:  (systematic[sb] OR meta-analysis[pt] OR meta-analysis as topic[mh] OR meta-analysis[mh] OR meta analy*[tw] OR metanaly*[tw] OR metaanaly*[tw] OR met analy*[tw] OR integrative research[tiab] OR integrative review*[tiab] OR integrative overview*[tiab] OR research integration*[tiab] OR research overview*[tiab] OR collaborative review*[tiab] OR collaborative overview*[tiab] OR systematic review*[tiab] OR technology assessment*[tiab] OR technology overview*[tiab] OR "Technology Assessment, Biomedical"[mh] OR HTA[tiab] OR HTAs[tiab] OR comparative efficacy[tiab] OR comparative effectiveness[tiab] OR outcomes research[tiab] OR indirect comparison*[tiab] OR ((indirect treatment[tiab] OR mixed-treatment[tiab]) AND comparison*[tiab]) OR Embase*[tiab] OR Cinahl*[tiab] OR systematic overview*[tiab] OR methodological overview*[tiab] OR methodologic overview*[tiab] OR methodological review*[tiab] OR methodologic review*[tiab] OR quantitative review*[tiab] OR quantitative overview*[tiab] OR quantitative synthes*[tiab] OR pooled analy*[tiab] OR Cochrane[tiab] OR Medline[tiab] OR Pubmed[tiab] OR Medlars[tiab] OR handsearch*[tiab] OR hand search*[tiab] OR meta-regression*[tiab] OR metaregression*[tiab] OR data synthes*[tiab] OR data extraction[tiab] OR data abstraction*[tiab] OR mantel haenszel[tiab] OR peto[tiab] OR der-simonian[tiab] OR dersimonian[tiab] OR fixed effect*[tiab] OR "Cochrane Database Syst Rev"[Journal:__jrid21711] OR "health technology assessment winchester, england"[Journal] OR "Evid Rep Technol Assess (Full Rep)"[Journal] OR "Evid Rep Technol Assess (Summ)"[Journal] OR "Int J Technol Assess Health Care"[Journal] OR "GMS Health Technol Assess"[Journal] OR "Health Technol Assess (Rockv)"[Journal] OR "Health Technol Assess Rep"[Journal]) AND (randomized controlled trial[pt] OR randomized controlled trials as topic[mh] OR random allocation [mh] OR double-blind method[mh] OR single-blind method[mh] OR random*[tw] OR "Placebos"[Mesh] OR placebo[tiab] OR ((singl*[tw] OR doubl*[tw] OR trebl*[tw] OR tripl*[tw]) AND (mask*[tw] OR blind*[tw] OR dumm*[tw])) AND ("humans"[mh] OR human*[tw])) |
| --- |
| Randomized controlled trials in humans:  (randomized controlled trial[pt] OR randomized controlled trials as topic[mh] OR random allocation [mh] OR double-blind method[mh] OR single-blind method[mh] OR random*[tw] OR "Placebos"[Mesh] OR placebo[tiab] OR ((singl*[tw] OR doubl*[tw] OR trebl*[tw] OR tripl*[tw]) AND (mask*[tw] OR blind*[tw] OR dumm*[tw])) AND ("humans"[mh] OR human*[tw])) |
| Observational study types in humans:  (((("Observational Studies as Topic"[Mesh] OR "Observational Study"[Publication Type]) OR observational stud*[tw] OR "Cohort Studies"[Mesh]) OR cohort stud*[tw] OR "Cross-Sectional Studies"[Mesh]) OR cross-sectional Stud*[tw] OR "Case-Control Studies"[Mesh] OR case-control Stud* OR "Ecological study"[tw] OR "Review Literature as Topic"[Mesh:noexp] NOT randomized controlled trial[pt] NOT random*[tw] AND ("humans"[mh] OR human*[tw])) |
| Environmental and plant studies:  OR "Periphyton"[mh] OR periphyton[tw] NOT animals [tw] NOT humans [tw] NOT “gut microbiota”[tw] NOT rat[tw] NOT mice[tw]) AND (("Environmental Microbiology"[mh] OR "Ecosystem"[mh] OR "Environment"[mh] OR "Natural Resources"[mh] OR "Atmosphere"[mh] OR "Extraterrestrial Environment"[mh] OR "Earth, Planet"[mh] OR biodiversity[tw] OR environment[tw] OR ecosystem[tw] OR water[tw] OR soil[tw] OR air[tw] OR earth[tw]) OR ("Plant Development"[Mesh] OR "Plant Growth Regulators"[Mesh] OR "Plant Structures"[Mesh] OR "Crops, Agricultural"[Mesh] OR "Plants"[Mesh] OR "Plants, Medicinal"[Mesh] OR "plant structure"[tw] OR "plant component"[tw] OR "cultivated Plants"[tw] OR plant[tw] OR herbal[tw] OR crop[tw]))) |
| Animal and laboratory studies:  ("Models, Biological"[Mesh:NoExp] OR "Disease Models, Animal"[Mesh] OR "animal model*"[tw] OR "Animal Experimentation"[Mesh] OR "Animal Experimentation"[tw] OR "Clinical Laboratory Techniques"[Mesh] OR (("veterinary"[Subheading] OR "veterinary"[All Fields]) AND Observational Study[ptyp])) |
| Other designs:  (((("Non-Randomized Controlled Trials as Topic"[Mesh]) OR Non-Randomized Controlled[tw] OR "Case Reports" [Publication Type]) OR “Case Report”[tw] OR "Consensus Development Conference" [Publication Type]) OR consensus[ti] OR conference[ti] NOT "Randomized Controlled Trial" [Publication Type] NOT randomized[tw]) |
| Commentaries, editorials, news items and letters:  ("Comment"[Publication Type] OR comment[tw]OR "Editorial"[Publication Type] OR editorial[tw] OR "Letter"[Publication Type] OR letter[tw]OR"News" [Publication Type] OR news[ti])  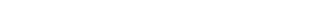 |
