# Supplementary material for: Microbiome research in general and business newspapers: How many microbiome articles are published and which study designs make the news the most?
Source: PLoS One. 2021 Apr 9;16(4):e0249835. doi: 10.1371/journal.pone.0249835 (PMC8034714; doi:10.1371/journal.pone.0249835)
Supplement: Attachment — Submitted filename: Response to Reviewers.docx [file pone.0249835.s004.docx]

| **Nº** | **Comments** | **Answer** |
| --- | --- | --- |
| Academic editor’s comments | | |
| Journal requirements | | |
| 01 | Please ensure that your manuscript meets PLOS ONE’s style requirements, including those for file naming. | Both new files ‘Revised Manuscript with Track Changes’ and ‘Manuscript’ meet PLOS ONE’s style requirements regarding title, author, affiliations and main body. We have also updated file naming for figures and for supporting information, which have been resubmitted to meet PLOS requirements. |
| 02 | Thank you for stating the following in the Competing Interests section:  "I have read the journal's policy and the authors of this manuscript have the following competing interests: APB is a paid consultant to companies commercially involved in the gut microbiota and probiotics. GC has declared no competing interests."  Please confirm that this does not alter your adherence to all PLOS ONE policies on sharing data and materials, by including the following statement: "This does not alter our adherence to  PLOS ONE policies on sharing data and materials.” (as detailed online in our guide for authors http://journals.plos.org/plosone/s/competing-interests).  If there are restrictions on sharing of data and/or materials, please state these. Please note that we cannot proceed with consideration of your article until this information has been declared.  Please include your updated Competing Interests statement in your cover letter; we will change the online submission form on your behalf. | We have included our updated Competing Interests statement in the cover letter so the online submission form may be changed on our behalf. |
| Reviewers’ comments: review comments to the author | | |
| Reviewer #1 | | |
| Major points | | |
| 01 | The exact and long-term aim of such analysis could be better explained. It is important for researchers to translate their results to the general public. What they can improve? | Thank you for your consideration.  We have changed the way we state the aim and scope of our analysis in the introduction and discussion of the manuscript. |
| 02 | Are the news coverages coming from the article press-releases? This is important to address. | Thank you for acknowledging this point. Although this objective has not yet been studied in the context of the microbiome, it has been subject to considerable study for different scientific and biomedical topics ([Entwistle, 1995](https://www.bmj.com/content/310/6984/920); [De Semir et al., 1998](https://jamanetwork.com/journals/jama/fullarticle/187769); [Stryker, 2002](https://www.sciencedirect.com/science/article/abs/pii/S0091743502911023); [Schwartz et al., 2012](https://www.bmj.com/content/344/bmj.d8164)). For instance, one of the manuscript’s authors has documented that journalistic articles in the Spanish press discussing current biomedical issues are intensely mediated by press releases ([Casino, 2015](https://www.tremedica.org/wp-content/uploads/n42_tribuna-GCasino.pdf)).  The study of microbiome-related press releases does not seem to be a priority at the moment and such an exercise will require another methodology that is outside the scope of our manuscript.  We have clarified in the introduction of the manuscript that the influence of press releases on news coverage of scientific and biomedical topics has been widely studied. |
| 03 | It would be interesting to analyze if the citations of the paper depend on press coverage. | Thank you for your observation. Previous studies cited in the manuscript’s introduction have shown that scientific articles that receive press coverage have, on average, more citations in the scientific literature compared to those not mentioned in the press.  Exploring to what extent citations of microbiome papers depend on press coverage is outside the scope of this manuscript.  In the introduction of the first submitted version of the manuscript, we cited studies (references 11-16) supporting the influence of press coverage on subsequent scientific citations. |
| 04 | The remarkable under-representation in the press of environmental & plant studies should be better explained in the discussion. A potential reason could be that environmental & plant studies are harder to apprehend in context of the microbiome to make reader-friendly stories in the press. (?) | Thank you for encouraging us to reflect on an explanation for the under-representation in the press of environmental & plant studies.  We have explained in the discussion that the remarkable under-representation of environmental & plant studies in the newspapers under analysis might have its roots in the fact that the level of knowledge required to understand and communicate their findings in layman’s terms is higher than that required for observational study types. In addition, environmental & plant studies are usually published in less prestigious journals that probably do not issue press releases. Third, the studies may be less newsworthy because do not have a direct impact on human health.  We have also acknowledged that the over-representation of observational study designs can distort the public’s perceived image of the microbiome, as those study design types are often inaccurately reported in newspapers and usually do not mention any associated caveats and limitations. |
| 05 | The limitations of the study by not including the impact of microbiome research in other media have been mentioned in the discussion. A short explanation of the methodological limitations using other types of media could underline the reason for the restricted methods with “only” six newspapers analyzed. | We are aware that we have not included any other mainstream media, social media or blogs in our analysis.  In response to your insightful comment, we have stated in the introduction that, although most people go online to search for information about scientific issues, newspaper content continues to dominate the online information repertoire over other media types.  Analyzing press citations has advantages over alternative metrics (known as altmetrics) for scientific publications in websites, blogs and social media. That is because the press is the main news producer and allows for reproducible tracking of newspaper articles on a specific topic within a defined period of time via the Factiva database, used in previous studies looking at the press coverage of biomedical research. That is not always possible for a content analysis of other types of media.  In addition, the selection of the sample of six newspapers responds to three patterns of reporting biomedical research in the press—USA, UK and Western World—described by the author Gonzalo Casino ([Casino et al., 2017](https://bmjopen.bmj.com/lookup/pmidlookup?view=long&pmid=29133334)), which has also been acknowledged in the introduction. As such, our sample selection of newspapers might be seen as an indicator of how other newspapers from the same area of the world report microbiome research. |
| Minor points | | |
| 01 | The visualization of filtering process in Fig.1 could be improved by highlighting the selected paths for analysis. | We agree with your suggestion for improving the visualization of the methodology used in our manuscript.  We have changed figure 1 of the manuscript and increased the width of the lines to highlight the selected paths that have been used in our data analyses. |
| Reviewer #2 | | |
| Major points | | |
| 01 | For this reviewer it is completely unclear how the authors evaluated the media coverage of certain articles. As far as I interpreted the reported data, the authors have classified articles available on PubMed regarding study evidence. I would prefer to have a comparison between top quality RCTs regarding media coverage. What was the reason for reports in the media? This would support scientists in getting their research recognized by lay audience via media coverage. | In an initial step, we downloaded all news stories on the microbiome (excluding opinion-editorial articles) and read all of them individually to identify the study mentioned, based on the author’s name, the name of the journal or by accessing the provided link to the scientific publication. Then, we proceeded with classifying the study design of the microbiome papers mentioned in the press and used study designs published in PubMed between 2007 and 2019 as a comparison group.  Analysis of the impact of study designs in the press is an active area of research that has been applied in the context of biomedical news, as stated in the methods section. We have adapted the criteria used by [Bartlett et al.](https://www.bmj.com/content/325/7355/81#:~:text=We%20conducted%20a%20longitudinal%20study,one%20broadsheet%20and%20one%20tabloid.) and [Lai and Lane](https://journals.plos.org/plosone/article?id=10.1371/journal.pone.0006103) for categorizing the study design in newspapers in the context of microbiome research (i.e., we have created a new category for environmental & plant studies due to the special relevance of this research in the microbiome field, according to [Stulberg et al](https://www.nature.com/articles/nmicrobiol201515).). As a result, in our analysis of newspapers and PubMed, we have considered the 6 study design categories defined in our manuscript as follows: SRs of RCTs, RCTs, observational study types, animal or laboratory studies, environmental & plant studies, and other designs.  In line with the criteria used by [Bartlett et al.](https://www.bmj.com/content/325/7355/81#:~:text=We%20conducted%20a%20longitudinal%20study,one%20broadsheet%20and%20one%20tabloid.) and [Lai and Lane](https://journals.plos.org/plosone/article?id=10.1371/journal.pone.0006103), and given that [previous work](https://journals.plos.org/plosone/article?id=10.1371/journal.pone.0145294) suggests that large RCTs that report hard outcomes (i.e. high-quality RCTs) usually attract the same press interest as low-quality RCTs, we have not focused solely on the press coverage of high-quality RCTs. However, our classification does differentiate between randomized controlled trials (high-quality RCTs) and intervention studies without randomization and/or without a control group (low-quality RCTs). The latter has been included in the “other designs” category, as stated in the methods section.  Study quality is evaluated after publication and such an analysis would have required a specific methodology (such as [the GRADE system](https://www.gradeworkinggroup.org/)), which is outside the scope of this study.  In the methods section, we have clarified the method used for classifying all of the different study design types mentioned in microbiome news stories. |
| 02 | What was the difference between the three countries and did the authors evaluate reasons for the observed differences? | Thank you for acknowledging this point.  First of all, we have clarified in the methods section that the three countries (USA, UK and Spain) were selected as they were representative of three [previously identified](https://bmjopen.bmj.com/content/7/11/e018705) national patterns of biomedical reporting in the press (American, British and Western World).  We have stated in the results section that American newspapers showed a greater interest in microbiome research, followed by British newspapers and, lastly, Spanish newspapers. When it comes to study design, no major differences were found between countries, as shown in figure 3B. However, at an individual level, *The New York Times* showed an over-representation of SRs of RCTs in humans, while *The Times* over-represented RCTs compared to PubMed.  We have also evaluated in detail in the discussion the reasons behind the observed differences. American and British newspapers’ considerable interest in microbiome research compared to the Spanish press might reflect the USA and the UK’s longer and more far-reaching scientific tradition and that tradition’s dominant position in the scientific literature. Second, British and American newspapers also echo the top medical journals the most. Third, the over-representation of SRs of RCTs and RCTs in *The New York Times* and *The Times*, respectively, may be because the USA and the UK are the countries that produce the most Cochrane SRs and newspapers tend to report more on domestically produced science. |
| 03 | There should be a fact box included on game changers regarding media coverage of scientific data. | We agree with the fact that it is important to dedicate special attention in our manuscript to changes in the landscape of coverage of scientific data with the emergence of social media and blogs as news sources.  In the introduction section, we have devoted several paragraphs to discussing online content and social media as new players involved in how scientific data is covered by the media. We also discuss social media’s impact on how the lay public engages with science, but also as a means by which scientists can improve their citations in academic journals. Although the lay public uses the internet as the main source for keeping up-to-date with scientific issues, newspaper content still dominates the online information repertoire and social media are used by newspapers as a way of amplifying their own content. |
| 04 | Was the media coverage accompanied by better funding of research groups? This would be essential information that could enhance the impact of the reported data. | Thank you for your comment on the relationship between the funding of research and media coverage of scientific articles.  There is little data in the literature regarding whether media coverage of scientific articles is used by funding entities to assign money for research and this objective is outside the scope of our manuscript.  Competitive research funding devotes part of the budget to communication and dissemination activities, which means more visibility in the media for the research group and thus greater probability of obtaining funding. However, media coverage is only one of the factors that can improve the funding of research projects.  As explained in the discussion, although mentions of scholarly outputs in social media and news sites are increasingly included in policy papers and research calls, it is too early to consider whether they contribute to the awarding of research funding. |
| 05 | Additionally the authors only report regarding newspapers, which is not targeting the younger population. What about social media, online publications,..? | We are aware that we have not included social media and other online publications in our analysis.  In the updated version of our manuscript, we have stated in the introduction that even though most people go online to search for information about scientific issues, newspaper content still dominates the online information repertoire over other types of media. In addition, the social media platforms used by the younger population tend to get their information from digital newspaper content. As we are aware of the changing landscape in which traditional media is moving from print to digital platforms, we included in our analysis the digital version of the newspapers under study when they were included in the Factiva database. |
| 06 | In the current form, the data of the manuscript might be better reported in a letter to the editor. | We appreciate the reviewer’s suggestion, but both authors consider that the results of this manuscript are original and novel and therefore are a better fit for the research article category. Furthermore, as far as we know, *PLOS ONE* does not include letters to the editor. |
| Minor comments | | |
| 01 | Fig. 1. Please specify what Op-Ed is. | Fig. 1 has been updated and includes the explanation of what Op-Ed is (i.e., opinion-editorial articles). |
| 02 | Please provide more information about SINC (country, city) and why this agency was chosen. | In the methods section (subsection “Newspaper coverage of microbiome research”), we have provided detailed information about SINC. |
