# Supplementary material for: Microbiome research in general and business newspapers: How many microbiome articles are published and which study designs make the news the most?
Source: PLoS One. 2021 Apr 9;16(4):e0249835. doi: 10.1371/journal.pone.0249835 (PMC8034714; doi:10.1371/journal.pone.0249835)
Supplement: Attachment — Submitted filename: Response to Reviewers.docx [file pone.0249835.s005.docx]

| **Nº** | **Comments** | **Answer** |
| --- | --- | --- |
| Academic editor’s comments | | |
| Journal requirements | | |
| 01 | Please review your reference list to ensure that it is complete and correct. If you have cited papers that have been retracted, please include the rationale for doing so in the manuscript text, or remove these references and replace them with relevant current references. Any changes to the reference list should be mentioned in the rebuttal letter that accompanies your revised manuscript. If you need to cite a retracted article, indicate the article’s retracted status in the References list and also include a citation and full reference for the retraction notice. | The reference list of the manuscript has been reviewed in detail. It does not contain papers that have been retracted nor unavailable and unpublished work and personal communications.  All the references included in the manuscript as it stands now meet criteria specified in PLoS One submission guidelines (<https://journals.plos.org/plosone/s/submission-guidelines#loc-references>). |
| Reviewers’ comments: review comments to the author | | |
| Reviewer #3 | | |
| Review comments to the author | | |
| 01 | Applying the exclusion criteria proposed by the Authors, the news related to microbioma without an explicit reference to scientific papers remain out of the analysis. This is not a problem per se, but if the Authors claim to study the "social impact" of the microbioma's research the generic discourse about it in the newspapers is a relevant aspect. As a consequence, it cannot be completely ignored and if the Authors decide to leave this aspect outside the paper, it should be discussed, at least. | Thank you for acknowledging this point as this question needs to be discussed in depth.  For our first objective of quantifying the six newspapers’ interest in the microbiome, we focused both on news stories on the microbiome that cite at least one scientific paper (361/518=69.7%) and news stories on the microbiome that do not reference a scientific paper (157/518=30.3%), in relation to the total number of news stories that had the microbiome as the main topic. For the second objective of quantifying which microbiome study design made the news the most, we only focused on news stories that explicitly mentioned a scientific publication on the microbiome because we wanted to quantitatively compare that to PubMed publications.  News stories on the microbiome that did not reference a scientific paper can be addressed according to a qualitative methodology that explores several variables, including the topics addressed and the language used to discuss the latest scientific advances in microbiome research. However, this research objective is outside the scope of our manuscript. It should also be acknowledged that other authors have focused on studying news stories on the microbiome from the perspective of language and by using a qualitative methodology ([Chuong KH et al. 2015](https://journals.sagepub.com/doi/10.1177/1049732314568199); [Nerlich & Hellsten, 2009](https://www.tandfonline.com/doi/abs/10.1080/14636770802670233); [Nerlich, 2017](https://blogs.nottingham.ac.uk/makingsciencepublic/2017/05/12/microbiome-goes-viral/)).  We have re-written the first part on newspaper coverage of microbiome research in the methods section to better clarify our workflow and analysis. We are aware that excluding news stories that had the microbiome as the central topic but which did not reference a scientific paper is a limitation of our manuscript and have acknowledged this point in the discussion. |
| 02 | Moreover, taking into account the above cited selection criteria, it is not surprising "the strong presence of research in the news stories about microbiome in the press". This result, indeed, mostly depends on precisely the application of such exclusion criteria. | Thank you for your comment.  The microbiome is a current hot topic for newspaper coverage and, in an initial step, we applied strict selection criteria to ensure that the news stories under analysis covered the microbiome as the central topic, in an in-depth and objective way.  If a news story mentioned the microbiome or any of its synonyms in a quote, for contextualizing a recipe or in the title of a book, we did not consider them to be relevant enough in terms of informing on the microbiome. Therefore, articles that mentioned the microbiome at some point but where the microbiome was not the focus—i.e. it was discussed in less than 50% of the text—were excluded from our analysis. This sampling process is known to eliminate news stories that contain tangential microbiome-related content (i.e., false positives) ([Guasch et al., 2019](https://ijoc.org/index.php/ijoc/article/view/9843/2580)).    In our first objective of quantifying the newspapers’ interest in the microbiome, we found that news stories on the microbiome that do not mention a scientific article represented a minority (30.3%), in relation to the total number of news stories that had the microbiome as the main topic. As we were interested in doing a controlled comparison between the scientific literature and newspapers, in our second objective, we ruled out news stories on the microbiome that did not mention a scientific paper in order to compare the same variable (mention of a scientific paper) in the six newspapers and in articles collected via PubMed.  We have acknowledged in the discussion the limitation of not considering all representations of the microbiome, regardless of whether they cite a scientific study, in the newspapers we analyzed. |
| 03 | It should be explained why the scientific papers most cited in the news have gained such a position. In other words, why these paper are more newsworthy than others? | Thank you for your observation.  There is no one factor alone that can explain why some microbiome scientific articles are more newsworthy than others. In our analysis we have shown that study design could influence journalists’ selection of the scientific papers that will be echoed in newspapers. Beyond study design, our decision to focus on influential newspapers in terms of readership and circulation might explain the intense coverage of scientific findings in the press, as these newspapers have large science and medicine sections with specialist reporters. Other factors that might also be important include the impact factor of the journal, the availability of press releases, the domestic preference of newspapers for journals from their own country, and the newsworthiness of the topic.  We have clarified in the discussion which factors, beyond study design, may explain why some microbiome scientific papers are more newsworthy than others (lines 351-362). To date, different partial analyses have been done studying which factors shape how scientific and medical findings are echoed in newspapers, while in future an overall analysis could clarify which factors have the strongest weight in determining which microbiome scientific papers are finally echoed in the media. |
| 04 | The Authors listed three reasons for explaining “the remarkable under-representation of environmental & plant studies in the newspapers”. The last is that this kind of “studies may be less newsworthy because do not have a direct impact on human health”, but I wonder whether this can be actually the first and the most relevant. | Thank you for encouraging us to reflect on this explanation for the under-representation of environmental & plant studies in the newspapers under analysis.  We agree on your point of view. Therefore, we have stated in the discussion that the fact that environmental & plant studies do not have a direct impact on human health might be the most relevant underlying factor in our findings and have placed it in first position on the list. Moreover, we have also cited the findings of two large population surveys that have shown that the topics of greatest interest to society are those of medicine and health, with scientific and technological discoveries and the environment and ecology generating far less interest ([National Science Board, 2018](https://www.nsf.gov/statistics/2018/nsb20181/report/sections/science-and-technology-public-attitudes-and-understanding/highlights); [Fundación Española para la Ciencia y la Tecnología (FECYT), 2018](https://icono.fecyt.es/sites/default/files/filepublicaciones/20/percepcion_social_de_la_ciencia_y_la_tecnologia_2018_completo_0.pdf)). |
